# A reanalysis of mouse ENCODE comparative gene expression data

**DOI:** 10.12688/f1000research.6536.1

**Published:** 2015-05-19

**Authors:** Yoav Gilad, Orna Mizrahi-Man

**Affiliations:** 1 Department of Human Genetics, University of Chicago, Chicago, IL, 60637, USA

**Keywords:** ENCODE, RNA-seq, developmental pathways, flowcells, sequencing

## Abstract

Recently, the Mouse ENCODE Consortium reported that comparative gene expression data from human and mouse tend to cluster more by species rather than by tissue. This observation was surprising, as it contradicted much of the comparative gene regulatory data collected previously, as well as the common notion that major developmental pathways are highly conserved across a wide range of species, in particular across mammals. Here we show that the Mouse ENCODE gene expression data were collected using a flawed study design, which confounded sequencing batch (namely, the assignment of samples to sequencing flowcells and lanes) with species. When we account for the batch effect, the corrected comparative gene expression data from human and mouse tend to cluster by tissue, not by species.

## Introduction

The mouse ENCODE Consortium has collected multiple types of genomic and functional data in order to better understand the potential utility of the mouse as a model system for biomedical research. To study gene expression levels, the Consortium collected RNA sequencing data from multiple tissues from human and mouse. Their comparative analysis revealed that gene expression patterns tend to support clustering of the data by species, rather than by tissue (Figure 2a in reference 1).

This pattern was confirmed and discussed in greater detail in a companion paper by Lin *et al.* ^2^, which also acknowledged that this observation is somewhat unexpected. Indeed, previous comparative studies reported that gene expression data from human and mouse (and across other species more generally) tend to cluster by tissues, not by species. Lin *et al.* proposed that previous studies might have been biased in their focus on a few ‘specialized’ tissues that tend to express the largest number of ‘tissue-specific genes’, while the overall pattern supports less tissue specificity.

The implications of the observation that human and mouse gene expression data may be clustering by species more than by tissues can be profound. To a large degree, modern biology is built upon the empirical observation that homologous gene regulatory networks establish the identities of homologous cell-types, tissues, and organs across species – the results of Lin *et al.*, if true, challenge these observations and the biological basis of homology. From a more practical perspective, the mouse is arguably the most important animal model for biomedical research. If gene regulation in any mouse tissue is markedly more representative of a general mouse regulatory network than the regulatory network of a corresponding human tissue, this would call into question the utility of the mouse, and perhaps any other non-human animal, as a useful model system for biomedical research.

Here, we present a reanalysis of the mouse ENCODE Consortium comparative RNA sequencing data. We argue that a flaw in their study design raises doubt regarding their conclusions.

## Methods

### RNA-Seq data, genome and gene annotation files

In December 2014 we asked and were kindly provided by the authors of Lin *et al.* ^2^ the names of the sequence files used in their comparative analysis. Based on this information we obtained sequence files in FASTQ format ( Supplementary Table 1) from the ENCODE project ^1^ site ( https://www.encodeproject.org/; some of the files were only available from early January 2015).

For our analysis, we used the same genome build and gene annotation files as in Lin *et al.* ^2^. The ENSEMBL ^3^ genome build *Mus musculus* GRCm38.68 was downloaded from ftp://ftp.ensembl.org/pub/release-68/fasta/mus_musculus/dna/Mus_musculus.GRCm38.68.dna_sm.toplevel.fa.gz; the corresponding transcript annotation file was downloaded from ftp://ftp.ensembl.org/pub/release-68/gtf/mus_musculus/Mus_musculus.GRCm38.68.gtf.gz. The *Homo sapiens* genome build provided by ENSEMBL ^3^ contains haplotypic regions that are not part of the primary assembly. To avoid these regions, genome build *Homo sapiens* GRCh37 was downloaded from the Illumina iGenomes page: ( http://support.illumina.com/sequencing/sequencing_software/igenome.html). The GENCODE ^4^ Release 14 transcript annotation file for human was downloaded from ftp://ftp.sanger.ac.uk/pub/gencode/release_14/gencode.v14.annotation.gtf.gz. The chromosome names in the GENCODE gtf file did not match those in the genome sequence file, and were thus modified.

### Sequencing study design

Based on the sequence identifiers found in the FASTQ files, we reconstructed the sequencing study design used to collect the gene expression data in Lin *et al.* ^2^. The sequence identifier line in a FASTQ file generated from an Illumina sequencing run can take two formats, depending on the version of the Consensus Assessment of Sequence and Variation (CASAVA) pipeline used to generate it. Prior to version 1.8 of this pipeline the sequence identifier line was of the following format (CASAVA v1.7 user guide p.88; downloaded from: http://support.illumina.com/downloads/casava_software_version_17_user_guide_(15011196_a).html

@<machine_id>:<lane>:<tile>:<x_coord>:<y_coord>#<index>/<read_#>

Starting from version 1.8 the sequence identifier line is of the format http://support.illumina.com/help/SequencingAnalysisWorkflow/Content/Vault/Informatics/Sequencing_Analysis/CASAVA/swSEQ_mCA_FASTQFiles.htm

@<machine_id>:<run number>:<flowcell ID>:<lane>:<tile>:<x-pos>:<y-pos> <read>:<is filtered>:<control number>:<index sequence>

Below is a sequence identifier line from the mouse pancreas read1 FASTQ file (sequence identifier lines from the remaining FASTQ files were of similar format):

@D4LHBFN1:276:C2HKJACXX:4:1101:3448:12374 1:N:0:AGTTCC

**Figure 1.**
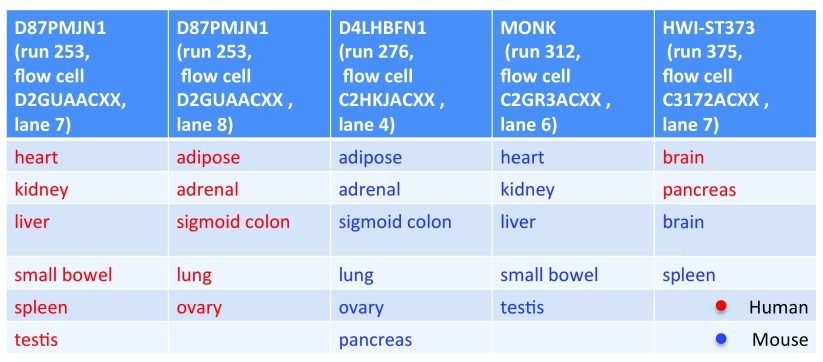
Study design. Sequencing batches as inferred based on the sequence identifiers of the RNA-Seq reads.

Based on this information we inferred that the FASTQ files were generated by CASAVA version 1.8 or higher. Thus, we could extract from the sequence identifiers the following details that pertain to the sequencing study design: machine identifier, run number, flowcell identifier, and flowcell lane number. We found that the sequencing was performed in five batches, each consisting of a multiplexed single run on a single lane on one of four sequencers ( Figure 1; note that two of the batches, composed of human samples only, differed only in their lane number). The design was such that only one batch contained samples from both species. The remaining four batches could be divided into pairs where each of the two batches had a nearly identical tissue composition, but a different species.

### Ortholog annotation

Following Lin *et al.* ^2^, we used the protein-coding ortholog list generated by the modENCODE and mouse ENCODE consortia ^5^. A file containing all orthologs from human, mouse, fly and worm was downloaded from http://compbio.mit.edu/modencode/orthologs/modencode.common.orth.txt.gz. From this list we extracted 14,744 human-mouse one-to-one ortholog pairs, for which both members were included in the transcript annotation files we used. We note that this number is lower than the ~15,106 ortholog pairs reported in Lin *et al.* We are not certain of the meaning of the ‘~’ in the report of the number of ortholog pairs analyzed by Lin *et al.* Nevertheless, we believe that a possible explanation for this disparity is a parsing error. The last two columns of the ‘modENCODE ortholog file’ represent the number of genes from each species in the ortholog group. One of the steps required to obtain the subset of ortholog groups for analysis is to select those records where the two last columns have a value of 1 (i.e. one-to-one ortholog pairs). We found that if this selection is done through a command line search that does not require that the value in the last column be exactly “1”, but rather just begins with “1”, then the result is 15,104 putative human-mouse ortholog pairs.

### Quality assessment of RNA-Seq data

We used the FastQC software v0.10.0 ( http://www.bioinformatics.babraham.ac.uk/projects/fastqc/) to assess the quality of the individual FASTQ files ( Supplementary Table 2– Supplementary Table 6). We were concerned by evidence for GC content bias and overrepresented sequences. To examine the latter in greater detail, we mapped the sequences overrepresented in at least one sample to the genome of the respective species, using BLAT searches ^6^ against the hg19 (human) and mm10 (mouse) assemblies at the UCSC genome browser site ( http://genome.ucsc.edu/) ^6^. We found that in both species many of the overrepresented sequences mapped perfectly to the mitochondrial genome ( Supplementary Table 3– Supplementary Table 6). For the mouse pancreas sample only, we also found many overrepresented sequences mapped to regions with rRNA repeats from the SSU-rRNA_Hsa and LSU-rRNA_Hsa families.

### Mapping RNA-Seq reads to genome sequences

We mapped the RNA-Seq reads to their respective genomes using Tophat v2.0.11 ^7^ with the following options: “--mate-inner-dist 200” (i.e. inner mate distance is 200nt, based on paired-end reads with length 100nt each and an insert size of 350-450nt ); “--bowtie-n” (i.e. the “-n” option will be used in Bowtie ^8^ in the initial read mapping stage); “-g 1” (i.e. multi-mapping reads will be excluded from alignment); “-m 1” (i.e. one mismatch is allowed in the anchor region of a spliced alignment); “--library-type fr-firststrand” (the libraries had been constructed using the Illumina TruSeq Stranded mRNA LT Sample Prep Kit ^2^). An exception was the mouse pancreas sample, for which the mapping process stalled consistently at the same stage. For this sample we used Tophat v1.4.1 ^8^ with the same options as above. Tophat requires a Bowtie ^8^ index. For human we used the Bowtie index that was packaged with the genome sequence in the file downloaded from the Illumina iGenomes page ( http://support.illumina.com/sequencing/sequencing_software/igenome.html). For mouse we built an index using the bowtie-build utility from Bowtie v2.2.1 (v 0.12.7 for the index used with Tophat v1.4.1).

### Calculating gene GC content

For each of the two species we used the appropriate GTF file to generate a table, which contains for each gene its ENSEMBL gene identifier its common name, and the GC content of the sequence covered by the union of the gene’s transcripts. To this end, we first generated a GTF file where overlapping exons from different transcripts of the same gene were merged into a single “exon” with the same sequence coverage, retaining the association with the gene identifier. Next, we computed the nucleotide content of the exons in this new GTF file using the ‘nuc’ utility from bedtools v2.17.0 ^9^. Finally, we computed the GC content for each gene identifier by summing the number of ‘G’ and ‘C’ nucleotides in its merged exons and dividing by the sum of counts of unambiguous nucleotides in these exons.

### Per-gene FPKM values

We used Cufflinks v2.2.1 ^10^ to compute fragments per kilo base of transcript per million (FPKM) values and aggregate them per gene. The only option used was “--library-type fr-firststrand”. For the required transcript annotation file (“-G” parameter) we used the GTF file for the respective species described in the “Genome and gene annotation files” section. We then generated a matrix of 14,744 by 26 FPKM values for each gene (in the ortholog table) and sample. While generating this table we noticed that some of the common gene names were associated with more than one ENSEMBL gene identifier. In some cases we determined that this was due to gene identifiers that have been retired from the ENSEMBL database ^3^ but were retained in the GTF file (27 and 64 retired identifiers for human and mouse, respectively). These retired identifiers were ignored when constructing the FPKM matrix. For the remaining such cases we incorporated the value from the first appearance of the common name.

### Per-gene raw fragment counts

To compute per gene raw counts from the alignment files produced by Tophat ^7^, we used the program featureCounts v1.4.4 ^11^ with the respective species’ GTF file specified in the “Genome and gene annotation files” section. For all runs we used the following options: “-p” - indicates that fragments rather than reads should be counted; “-C” - indicates that chimeric fragments will not be included in the summarization process; and “-s 2” - indicates that the paired-ends are reversely stranded. We next generated a matrix of 14,744 by 26 raw counts for each gene (in the ortholog table) and sample. Since the output from featureCounts identifies genes by their gene identifier (the ENSEMBL identifier in our case), whereas the ortholog table uses the gene’s common name to identify it, we used the GC content table, which contains both these identifiers to match counts to the correct row in the ortholog table. As we did when generating the FPKM matrix, we ignored the values from retired ENSEMBL identifiers, and if there were still multiple identifiers for the same common name, we used the value from the identifier that appeared first.

## Results

In this reanalysis effort, we focused solely on the RNA sequencing data that can be mapped to coding regions. Lin *et al.* ^2^ reported additional results, related to data on the expression of non-coding transcripts and histone marks. We did not reanalyze these additional data types.

Lin *et al.* ^2^ analyzed both previously published and newly collected human and mouse gene expression data. The previously published data consist of RNA sequencing from ENCODE, the Illumina Human BodyMap 2.0, and the Roadmap Epigenomics Mapping Consortium. In these previously collected data sets, human and mouse samples were analyzed by different labs at different times, such that there is a clear batch effect that is confounded with species. Lin *et al.* ^2^ clearly explains this limitation of the previously published data. They state that in order to address this issue they focus on the analysis of only the newly collected data – RNA sequencing data of samples from 13 human and mouse tissues that were collected by the same lab, using the same sample processing protocol. We focus our reanalysis study on the same newly collected data set (see Methods).

### Replication of sample clustering by species

As a first step of our study we set out to replicate the analysis of Lin *et al.* ^2^. To do so, we started with the matrix of FPKM values (computed, using Cufflinks ^10^, based on the read alignments to the genome). This analysis was done within R environment v 3.1.3 GUI 1.65 Snow Leopard build (6912) ^12^. See Supplementary Text 1 for detailed commands, and a supplement zip file for the R input (available in Zenodo: http://dx.doi.org/10.5281/zenodo.17606).

**Figure 2.**
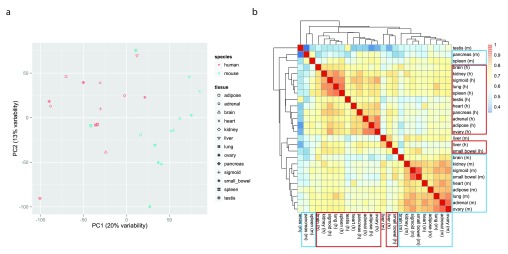
Recapitulating the patterns reported by the mouse ENCODE papers. **a**. Two-dimensional plots of principal components calculated by performing PCA of the transposed log-transformed FPKM values (from 14,744 orthologous gene pairs) for the 26 samples, after removal of invariant columns (genes). **b**. Heatmap based on pairwise Pearson correlation of expression data used in panel **a**. We used Euclidean distance and complete linkage as distance measure and clustering method, respectively.

We log _2_-transformed the FPKM matrix (after adding 1 to avoid undefined values). To visualize the data, we used an approach that is similar in principle to that used by the ENCODE mouse consortium and Lin *et al.* Specifically, we used the function ‘prcomp’ (with the ‘scale’ and ‘center’ options set to TRUE) to perform principal component analysis (PCA) of the transposed FPKM matrix (so that samples were now in rows and genes in columns), after removal of invariant columns (genes). Scatter plots of the PCA results were generated using the ggplot2 package ^13^. In agreement with the findings of Lin *et al.* ^2^ the samples cluster mostly by species ( Figures 2a, Figure S1 and Figure S2). We also plotted the heatmap of the matrix of Pearson correlations between the 26 samples, using the pheatmap function from the pheatmap package v1.0.2 ^14^ with default settings (i.e. complete linkage hierarchical clustering using the Euclidean distances). Again, samples from the same species tend to cluster together ( Figure 2b).

### Analysis of normalized data after accounting for batch effects yields clustering by tissue

A previous evaluation of normalization methods for RNA-Seq data ^15^ suggested that FPKM values were not optimal for clustering analysis. Therefore, as a basis for our reanalysis, we used the matrix of per-gene raw fragment counts. The entire analysis was done within R environment v 3.1.3 GUI 1.65 Snow Leopard build (6912) ^12^. See Supplementary Text 2 for detailed commands, and a supplement zip file for the R input (available in Zenodo: http://dx.doi.org/10.5281/zenodo.17606).

Following Li *et al.* ^16^, we removed the 30% of genes with the lowest expression as determined by the sum of fragment counts across all samples. Next, due to the presence of mitochondrial genes among the overrepresented sequences in the data, we also removed reads that map to the 12 mitochondrial genes. This left us with expression data from 10,309 genes for analysis. We note that merely limiting the analysis to this subset of genes does not have a marked effect on the patterns reported by Lin *et al.* ( Figure S3; detailed commands in Supplementary Text 3, and a supplement zip file for the R input (available in Zenodo: http://dx.doi.org/10.5281/zenodo.17606)). We performed within-column normalization to remove the GC bias in the data, indicated by the initial quality assessment. To this end, we applied the ‘withinLaneNormalization’ function from the EDASeq package v2.0.0 ^17^ to each column in the matrix, using the gene GC values for the species associated with the column. Next, we used the ‘calcNormFactors’ from the edgeR package v3.8.6 ^18^, with the trimmed mean of M-values (TMM) method ^19^, to calculate normalization factors for the library sizes for the samples. We used these normalization factors in the depth normalization of the columns (using the column sums of the original, unfiltered, counts matrix as a proxy for library sizes). The normalized data were log _2_-transformed (after adding ‘1’ to each value in the matrix to avoid undefined values).

We then considered how to account for the fact that the assignment of samples to sequencing flowcells and lanes was nearly completely confounded with the species annotations of the samples ( Figure 1). The consideration of ‘batch effect’ was the most important difference between the analysis that recapitulated the patterns reported by the mouse ENCODE papers (the previous ‘Results’ section) and the current reanalysis effort. Specifically, we accounted for the sequencing study design batch effects using the ‘ComBat’ function from the sva package v3.12.0 ^20^, with a model that includes effects for batch, species and tissue. For this purpose the samples were classified into five batches, based on the sequencing study design (see methods and Figure 1).

**Figure 3.**
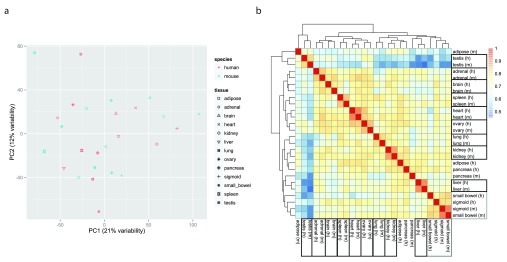
Clustering of data once batch effects are accounted for. **a**. Two-dimensional plots of principal components calculated by applying PCA to the transposed matrix of batch-corrected log-transformed normalized fragment counts (from 10,309 orthologous gene pairs that remained after the exclusion steps described in the results) for the 26 samples, after removal of invariant columns (genes). **b**. Heatmap based on pairwise Pearson correlation of the expression data used in panel a. We used Euclidean distance and complete linkage as distance measure and clustering method, respectively.

To visualize the data, we used the function ‘prcomp’ (with the ‘scale’ and ‘center’ options set to TRUE) to perform principal component analysis (PCA) of the transposed log-transformed matrix of ‘clean’ values (after removal of invariant columns, i.e. genes), and the ggplot2 package ^13^ to generate scatter plots of the PCA results. None of the first five principal components (accounting together for 56% of the variability in the data) support the clustering of the gene expression data by species ( Figure 3a and Figure S4– Figure S5). However, the sixth principal component, which accounts for 6% of the variability in the data, does support such a clustering, suggesting that even though the ‘species’ and ‘batch’ variables are confounded, accounting for ‘batch’ does not remove completely the variability due to ‘species’ ( Figure S5). We also plotted a heatmap of the matrix of Pearson correlations between the 26 samples, using the pheatmap function from the pheatmap package v1.0.2 ^14^ with default settings (i.e. complete linkage hierarchical clustering using the Euclidean distances). This time the heatmap shows considerable clustering of the comparaive gene expression data by tissue ( Figure 3b).

## Discussion

In our reanalysis we have made a number of specific choices, including the exclusion of a certain subset of lowly expressed genes, the specific approach we chose to summarize the count data, the standardization and normalization methods we used (for example, we chose to standardize by the total count of reads that mapped to the ortholog gene pairs), the approach we used to account for the GC content bias, and the method we used to account for the sequencing design batch effect. Moreover, we excluded the sequencing data from 12 mitochondrial genes from both species, a step that – to the best of our ability to determine – was not taken by the original studies. In addition, our definition of ortholog gene pairs differs slightly from that of the original study, as we discussed in the methods. In practice, only the correction for the sequencing design batch effect had a drastic impact on the results. For example, without accounting for batch, using per-gene raw fragment counts instead of FPKM values does not seem to impact the degree to which the uncorrected data support clustering by species ( Figure S6).

Visualizing or plotting the data is another important area where different choices can sometime lead to quite distinct conclusions. We chose to display, in addition to the PCA plots, heatmaps based on the correlations among the samples. We note that if the actual data (not pairwise correlations) are clustered, the observed patterns (by species or by tissues, in the respective analyses), seem practically identical ( Figure S7). The heatmaps shown in the main figures are based on Pearson pairwise correlations, which provide the highest level of clustering by tissue in the analysis that takes into account batch effects. Alternative heatmap plots based on either Spearman pairwise correlations or other distance measures and clustering methods look similar in principle ( Figure S8 to Figure S10), but the clustering by tissue is somewhat less pronounced (clustering by species, when batch is not accounted for, is more pronounced).

It is important to note that most of the analysis and plotting decisions we have made contributed to a somewhat better clustering of the expression data by tissue, both visually and empirically. We have made these – mostly standard - analysis and plotting choices regardless of the end result (namely, we believe that these are objectively reasonable choices). Importantly, we made identical choices for the clustering analysis and plot types for the data with and without batch correction, and our conclusions are robust with respect to a wide range of possible alternative approaches ( Figure S7– Figure S10).

That said, we do acknowledge that we find the clustering of the data by tissue to be a more intuitive pattern. In other words, we believe that the clustering of comparative gene expression data by species – a result that contradicts previous observations – is a surprising outcome. Hence, we would have intuitively accepted as more correct most reasonable choices of analysis pipelines and data visualizations that supported a greater degree of clustering by tissue.

As we mentioned above, most of the choices we made resulted in little difference to the overall pattern. It was only the correction for the sequencing design batch effect that had a profound impact. Once we accounted for the batch effect by using ComBat, the comparative gene expression data no longer clustered by species, and instead, we observed a clear tendency for clustering by tissue. This is not surprising, as the sequencing batch, which we corrected for, was nearly entirely confounded with species. It stands to reason that some individual gene expression levels do cluster by species and some by tissue (see for example, Figure S5). While previous data sets strongly support a general clustering of gene regulatory phenotypes by tissue ^21^, we expect the degree of clustering of the gene expression data to differ somewhat across tissues. Yet, in this particular case, by removing the confounding sequencing batch effect we also removed most of the species effect on gene expression levels (a similar case of confounding batch and main effect of interest was discussed a few years ago, with respect to gene expression differences between human populations ^22^).

One could potentially employ more sophisticated modeling approaches to try and estimate separately the batch and species effects. One idea would be to rely on the fact that there are five sequencing batches, but only two species. This, however, is complicated by the fact that the two sequence batches specific to the human samples share the same run and flowcell (potentially a smaller batch effect), while the two sequence batches specific to the mouse samples are extend over different instruments (potentially a larger batch effect). In any case, we feel that such modeling is beyond the scope of this reanalysis effort. Instead, we conclude that the study design used by the mouse ENCODE consortium was flawed with respect to the questions they set out to address.

In summary, we believe that our reanalysis indicates that the conclusions of the Mouse ENCODE Consortium papers pertaining to the clustering of the comparative gene expression data are unwarranted. In the narrow context of our reanalysis effort, we state that their conclusions are unwarranted, not wrong, because the study design was simply not suitable for addressing the question of ‘tissue’ vs. ‘species’ clustering of the gene expression data. That said, a large body of independent previous work supports general clustering of comparative gene expression data by tissue.

Finally, we note that in this reanalysis effort, we have only focused on the RNA sequencing data collected by the mouse ENCODE consortium. We have not considered information with respect to the study design used to collect the many other types of data reported by this consortium. Given our findings, we believe that it is appropriate to call for a careful review of these other data sets as well.

## Data availability

The data referenced by this article are under copyright with the following copyright statement: Copyright: ï¿½ 2015 Gilad Y and Mizrahi-Man O

Data associated with the article are available under the terms of the Creative Commons Zero "No rights reserved" data waiver (CC0 1.0 Public domain dedication). 

All data are available from the Mouse ENCODE consortium; see Table S1 for specific source URLs and accession numbers.

## Software availability

We provide supplementary files of the python codes used to process and prepare the data for analysis with R, and the data files for the python codes. We also provide the R codes we used to perform the different analyses as supplementary files, as well as the input for the R codes.

### Archived software files as at the time of publication

Zenodo. Data files and codes used in the reanalysis of the mouse encode comparative gene expression data. DOI: 10.5281/zenodo.17606

### License

These codes are provided under the MIT license.

## Supplementary tables

**Table S1.** Source of RNA-Seq data.

**Table S2.** Summary of test scores for the 52 FASTQ files analyzed.

**Table S3.** Overrepresented sequences in read1 human files.

**Table S4.** Overrepresented sequences in read2 human files.

**Table S5.** Overrepresented sequences in read1 mouse files.

**Table S6.** Overrepresented sequences in read2 mouse files.

## Supplementary figures

https://f1000researchdata.s3.amazonaws.com/supplementary/6536/43196de3-53a7-4a8f-9093-71ad32f461e4.pdf
