# Supplementary material for: A reanalysis of mouse ENCODE comparative gene expression data
Source: F1000Res. 2015 May 19;4:121. [Version 1] doi: 10.12688/f1000research.6536.1 (PMC4516019; doi:10.12688/f1000research.6536.1)
Supplement: Peer review history

# Peer review: Reviewer response for version 1

**DOI:** 10.5256/f1000research.7019.r8710

**Published:** 2015-06-30

**Authors:** Lior Pachter

**Affiliations:** 1 Department of Mathematics, University of California, Berkeley, Berkeley, CA, USA

The article "A reanalysis of mouse ENCODE comparative gene expression data" by Gilad and Mizrahi-Man examines a claim, recently published in the pair of papers

1. Yue F, Cheng Y, Breschi A, *et al*.: A comparative encyclopedia of DNA elements in the mouse genome. Nature. 2014; 515(7527): 355–364.
2. Lin S, Lin Y, Nery JR, *et al*.: Comparison of the transcriptional landscapes between human and mouse tissues. Proc Natl Acad Sci U S A. 2014; 111(48): 17224–17229.

that expression data from human and mouse cluster more by species than by tissue.

The Gilad--Mizrahi-Man paper consists of three "results":

1. A report of the experimental design in Lin *et al*. 
2. An attempt to reproduce the results of Yue *et al. *and  Lin *et al*. that pertain to the claim about species vs. tissue clustering of expression data.
3. A re-analysis of the Lin *et al. *data in a manner that addresses shortcomings in the original experimental design.

The first is the observation that Lin *et al*. improperly designed their experiment by confounding species with batches sequenced, thereby leading to a possible "batch effect" affecting their results. This observation was already published as a preprint by the first author on the pre-print server Twitter (see https://twitter.com/Y_Gilad/status/593088451462963202). 

Having noted "a flaw in their [Lin *et al*.] study design" Gilad--Mizrahi-Man turn to the question of whether the flaw affected the conclusions in Yue *et al*. and Lin *et al*. about expression differences as pertaining to tissues vs. species. To this end, the authors attempted to reproduce the analysis of Lin *et al*.

It is evident that while the Lin *et al*. results may, in some technical sense, be "reproducible" they were certainly not "usable" as published. Gilad--Mizrahi-Man carefully expose a vast number of choices in software and processing options poorly described in Lin *et al*., and whose effect on the final result(s) is unclear. To quote just one example, they write that "An exception was the mouse pancreas sample, for which the mapping process stalled consistently at the same stage", a problem that led them to use TopHat v1.4.1 instead of TopHat v2.0.11. One may wonder whether software choices and other decisions in analysis affect final results, and Gilad--Mizrahi-Man address this question (although only partly).

For example, one fundamental analysis choice is whether to quantify abundances of genes by summing raw "fragment counts" from alignments to gene regions, or via the summing of abundances as quantified by probabilistic assignment of ambiguously mapped reads. Gilad--Mizrahi-Man cite a paper by Dillies *et al*. (and the French StatOmique Consortium) suggesting that "FPKM values were not optimal for clustering analysis" to argue for using "fragment counts". I strongly disagree with this choice because transcript abundances are necessary to accurately estimate gene-level abundances, a point that Dillies *et al. *fail to realize. As pointed out in my own paper on Cufflinks 2 ( Trapnell *et al. *2012) wrong does not cancel wrong for differential analysis, nor does it for the purpose of clustering.

In any case, Gilad--Mizrahi-Man do examine whether quantification by EM affects results and in a later statement they state that "using per-gene raw fragment counts instead of FPKM values does not seem to impact the degree to which the uncorrected data support clustering by species", a result summarized in their Figure S6. While I applaud them for checking the dependence of results on this choice, without further analysis the question remains of whether other analysis choices affect results (although to be fair to the authors, the number of tests that would have to be conducted is enormous and quite possibly practically intractable). Nevertheless, it would be interesting if, for the purpose of future transcriptomics analyses, Gilad--Mizrahi-Man were to investigate some key steps as to their effect (e.g. annotation, an issue discussed recently by Ongen and Dermitzakis, or mapper choice).

The final result of Gilad--Mizrahi-Man is a re-analysis of the Lin *et al*. data from which they observe that a basic correction for batch effect removes the strong clustering of expression profiles by species touted in Yue *et al*. and Lin *et al. *The question of the relative species/tissue contribution to expression profile is of course fundamental and interesting, and obviously further data, carefully curated and analyzed, will answer the question definitively. As far as the Lin *et al. *paper goes, the Gilad--Mizrahi-Man paper certainly casts doubt on the suitability of the data for answering the question. For one thing, the term "batch effect" is unfortunately rather generic and in this specific case that has become a problem. After initial posting of the preprint by Gilad on Twitter, the authors of Lin *et al*. resequenced their libraries in a different configuration, but further investigation of the experimental design by Gilad *et al*. (subsequent to initial posting of a preprint of the article I'm reviewing) appears to have revealed additional problems. For example, tissues in human and mouse were selected from males vs. females respectively (except in ovaries and testis) resulting in another potential bias that would skew expression profiles to cluster by species rather than tissue. In other words, with their paper and subsequent analysis Gilad and Mizrahi-Man have convinced me to be skeptical of the data and conclusions of Lin *et al*. (and insofar as it pertains to the results in Lin *et al*., the paper by Yue *et al*.).

But that is not really the point. What matters now are the carefully documented serious shortcomings in the computational and experimental methodology of Lin *et al*. and for this reason I have approved the Gilad--Mizrahi-Man manuscript. Hopefully the issues raised will be properly addressed by Lin *et al*. (in a manner equally rigorous to that of Gilad--Mizrahi-Man).

Reviewer Expertise:

NA

I confirm that I have read this submission and believe that I have an appropriate level of expertise to confirm that it is of an acceptable scientific standard.

# Peer review: Reviewer response for version 1

**DOI:** 10.5256/f1000research.7019.r8832

**Published:** 2015-06-25

**Authors:** Mick Watson

**Affiliations:** 1 The Roslin Institute, University of Edinburgh, Edinburgh, UK

The study is carried out well and the results support the conclusion.

The paper would benefit from including some of the discussion points made in the comments made to v1 of the paper. Lin *et al. *have re-sequenced the samples and removed the sequence lane batch effect, and reproduced the same result; however, the samples themselves are confounded, in that they were treated differently prior to sequencing. This discussion should be added to the paper.

I would also like to see a discussion of artifacts which are discoverable within the data, for example:

- the human samples have significant numbers of rRNA reads compared to the mouse samples.  This should not happen with mRNA-Seq which includes a polyA selection.
- the human samples have a hugely varied number of reads per sample, compared to the mouse samples
- one of the mouse samples has over 1.8M reads that map to a single rRNA transcript. This is an outlier for mouse, as the other mouse samples have low numbers of rRNA reads
- The mitochondrial genes are turned on in one species but not the other

These points are all indicators of different sample extraction techniques, which also confound with species.

The authors may also wish to discuss use of FPKM, which may not be the most useful measure of gene expression in this study, as the human and mouse orthologues have different lengths.

See https://haroldpimentel.wordpress.com/2014/05/08/what-the-fpkm-a-review-rna-seq-expression-units/

Reviewer Expertise:

NA

I confirm that I have read this submission and believe that I have an appropriate level of expertise to confirm that it is of an acceptable scientific standard.

# Peer review: Reviewer response for version 1

**DOI:** 10.5256/f1000research.7019.r8942

**Published:** 2015-06-22

**Authors:** Michael Eisen

**Affiliations:** 1 Howard Hughes Medical Institute, University of California, Berkeley, Berkeley, CA, USA

In this paper Gilad and Mizrahi-Man reanalyze a high-profile dataset from the ENCODE consortium that was used to argue that gene expression levels are more similar for different tissues from the same species than the same tissues from different species, a somewhat counterintuitive result that contradicts earlier claims (including those by Gilad).

The main result of this new work is a simple observation: in the original experiment described in Lin *et al*., samples from the same species were run in the same sequencing batch. Since there are well-known batch effects, this is poor experimental design that calls into question the claim by Lin *et al*. that data clusters by species, since the data could instead by clustering by sequencing batch.

There is always a bit of a challenge in figuring out what to do with an observation. As the authors point out, this aspect of the experimental design effectively renders the data useless for asking questions about the relative contribution of species and tissue to gene expression variation. Yet it would seem like too light of a paper to simply say "The original authors messed up. Their claims are therefore invalid. QED." 

So this paper contains a few analyses designed to ILLUSTRATE the point they make. They are not exactly results, since, once you realize that batch and species are completely confounded, correcting for batch will inevitably remove the species signal. In this context, it's a bit weird to present such an analysis as if it is a result, but I don't really see a way around it, and the authors are forthright in pointing out that their main observation is that the data from Lin *et al *are useless for addressing this issue, and that they can make no claims, even after correcting for batch effects, about what the data actually do say.

Reviewer Expertise:

NA

I confirm that I have read this submission and believe that I have an appropriate level of expertise to confirm that it is of an acceptable scientific standard.

# Peer review: Reviewer response for version 1

**DOI:** 10.5256/f1000research.7019.r8732

**Published:** 2015-05-26

**Authors:** Rafael A. Irizarry

**Affiliations:** 1 Department of Biostatistics, Harvard School of Public Health, Boston, USA

In this PNAS paper is is found that the first three principal components obtained from mouse and human gene expression data correlate with species and not with tissue. This is interpreted to imply that "tissues appear more similar to one another within the same species than to the comparable organs of other species".

Gilad and Mizrahi-Man (the authors) downloaded all the data from this paper and reanalyzed it carefully. The majority of their F1000Research article is dedicated to describing, in full detail, how they analyzed the data. The choices made all seem sound and they are able to reproduce the figures of the original PNAS article.

An important discovery made and reported by the authors is that mice and human samples were run in different lanes or different instruments. The confounding was near perfect (see Figure 1). The authors then apply a linear model (ComBat) to account for the batch effect and find that, after the correction, samples cluster almost perfectly by tissue (see Figure 3). They conclude that "Once we accounted for the batch effect by using ComBat, the comparative gene expression data no longer clustered by species, and instead, we observed a clear tendency for clustering by tissue. This is not surprising, as the sequencing batch, which we corrected for, was nearly entirely confounded with species."

There are three issues I recommend the authors consider:

1. As the authors suggest, with the observed level of confounding, if there is in fact a species effect, applying an approach that models batch as a linear effect will also account for species. Although pointing out that there is almost perfect confounding is an important contribution, I don't see why ComBat should be applied here. If a model that removes species is applied, it is no surprise that the data will no longer cluster by species.
2. As mentioned, with the existing study design it is impossible to completely tease out species from batch. However, there is a relatively simple data analysis that can be performed to explore the possibility that instrument or lane are a large enough source of variability to overcome the tissue effect, which is know to be large. The analysis is:
  i) perform the same PCA analysis on the mouse data and compare the two instruments and then
  ii) perform PCA analysis on the human data and compare the two lanes.
  If in fact lane and instrument are a large sources of variability we should see it here. Of course, there is still the possibility that the instruments used for humans was very different to the one used for mice, while the two instruments used for mice were similar. Due to confounding we won't know for sure, but the analysis described here will at least give us at least a lower bound on how large these effects can be.
3. There is a comment in the F1000Research article from the first author of the PNAS article describing a second experiment in which confounding with instrument or lane was not present. In this analysis species continues to be the first few PCs. In a second version of this article, the authors can perhaps comment on this, as well as some of the other comments that suggest other possible sources of variability that may be confounded with species.

As a final remark, I am interested in reading the authors/readers thoughts on the biological interpretations that are being assigned to mathematical (euclidean) distance. Specifically, what does the word "similar" mean exactly. I understand what means in mathematics, but I am not sure what it means in biology when points are log (FPKM + 1) values for thousands of genes.

Reviewer Expertise:

NA

I confirm that I have read this submission and believe that I have an appropriate level of expertise to confirm that it is of an acceptable scientific standard, however I have significant reservations, as outlined above.

# Response

**Published:** 2015-05-26

**Authors:** Yoav Gilad

**Affiliations:** University of Chicago, USA

Dr. Irizarry,

Thank you for spending the time to provide a review of our work. We agree with you that given the study design used by the mouse ENCODE consortium, applying a batch correction is futile. Indeed, we explicitly explain that in our discussion (you referred to that section of the text in your review).

We further agree that it would be intellectually interesting to research the extent of the batch effect further – for example, by following your suggestion on how to test for the effect of instrument and lane.

However, we feel that this additional effort is beyond the scope of our study. The mouse ENCODE consortium papers did not discuss (or account for) the sequencing study design. **We spent considerable effort tracking the details that allowed us to reconstruct their design**. We pointed out in our paper that given this study design, the unusual biological result reported by the mouse ENCODE consortium might have a technical explanation. We believe it is the responsibility of the mouse ENCODE consortium authors to provide evidence that excludes this technical possibility, rather than us having to prove that it is indeed the likely explanation.

Which leads us to your third point: Indeed, the mouse ENCODE consortium authors commented that they have now collected additional sequence data, using a different design, and that their results held. In that sense, we believe that this means that the mouse ENCODE consortium authors accepted our claim that their original design was flawed.

Yet, as mentioned in a few other comments here, there is an additional technical batch effect that was not yet excluded – related to tissue extraction and sample preparation. We plan to discuss this additional technical batch effect in a revised version of the text (we will wait to see additional reviews before we provide a revised version of the paper).

Again, thank you for your time and thoughts.
